# The importance of CXCR4 expression in tumor stroma as a potential biomarker in pancreatic cancer

**DOI:** 10.1186/s12957-023-03168-6

**Published:** 2023-09-12

**Authors:** Raquel Bodoque-Villar, David Padilla-Valverde, Lucía María González-López, José Ramón Muñoz-Rodríguez, Javier Arias-Pardilla, Clara Villar-Rodríguez, Francisco Javier Gómez-Romero, Gema Verdugo-Moreno, Francisco Javier Redondo-Calvo, Leticia Serrano-Oviedo

**Affiliations:** 1 Traslational Investigation Unit, University General Hospital of Ciudad Real, SESCAM, Ciudad Real, Spain; 2 Research Institute of Castilla-La Mancha (IDISCAM), Ciudad Real, Spain; 3 Department of Surgery, University General Hospital of Ciudad Real, SESCAM, Ciudad Real, Spain; 4 Faculty of Medicine, University of Castilla-La Mancha, Castilla La Mancha, Ciudad Real, Spain; 5 Department of Pathology, University General Hospital of Ciudad Real, SESCAM, Ciudad Real, Spain; 6 Head of Research, University General Hospital of Ciudad Real, SESCAM, Ciudad Real, Spain; 7 Department of Anesthesiology, University General Hospital of Ciudad Real SESCAM, Ciudad Real, Spain

**Keywords:** Pancreatic ductal adenocarcinoma, CXCR4/CXCL12, Chemokines, Immunohistochemistry

## Abstract

**Background:**

Pancreatic ductal adenocarcinoma (PDAC) is one of the main causes of cancer mortality in the world. A characteristic feature of this cancer is that a large part of the tumor volume is composed of a stroma with different cells and factors. Among these, we can highlight the cytokines, which perform their function through binding to their receptors. Given the impact of the CXCR4 receptor in the interactions between tumor cells and their microenvironment and its involvement in important signaling pathways in cancer, it is proposed as a very promising prognostic biomarker and as a goal for new targeted therapies. Numerous studies analyze the expression of CXCR4 but we suggest focusing on the expression of CXCR4 in the stroma.

**Methods:**

Expression of CXCR4 in specimens from 33 patients with PDAC was evaluated by immunohistochemistry techniques and matched with clinicopathological parameters, overall and disease-free survival rates.

**Results:**

The percentage of stroma was lower in non-tumor tissue (32.4 ± 5.2) than in tumor pancreatic tissue (67.4 ± 4.8), *P*-value = 0.001. The level of CXCR4 expression in stromal cells was diminished in non-tumor tissue (8.7 ± 4.6) and higher in tumor pancreatic tissue (23.5 ± 6.1), *P*-value = 0.022. No significant differences were identified in total cell count and inflammatory cells between non-tumor tissue and pancreatic tumor tissue. No association was observed between CXCR4 expression and any of the clinical or pathological data, overall and disease-free survival rates. Analyzing exclusively the stroma of tumor samples, the CXCR4 expression was associated with tumor differentiation, *P*-value = 0.05.

**Conclusions:**

In this study, we reflect the importance of CXCR4 expression in the stroma of patients diagnosed with PDAC. Our results revealed a high CXCR4 expression in the tumor stroma, which is related to a poor tumor differentiation. On the contrary, we could not find an association between CXCR4 expression and survival and the rest of the clinicopathological variables. Focusing the study on the CXCR4 expression in the tumor stroma could generate more robust results. Therefore, we consider it key to develop more studies to enlighten the role of this receptor in PDAC and its implication as a possible biomarker.

## Backgrounds

Pancreatic cancer specifically pancreatic ductal adenocarcinoma (PDAC) is one of the main causes of cancer mortality in the world and its incidence has increased dramatically in recent decades. The 5-year survival rate does not exceed 10% and only 20% of patients have a surgical treatment option at the time of diagnosis [1].

A characteristic feature of PDAC tumors is that a large part of the tumor volume is composed of a tumor microenvironment, non-tumorous stroma, which accounts for more than 80% [2]. The tumor microenvironment is composed of tumor cells, innate and adaptive immune cells, fibroblasts, vascular and lymphatic endothelial cells, extracellular matrix, growth factors, hormones, protease, cytokines, and chemokines, among others [3]. Under normal conditions, these components function as a matrix that maintains homeostasis and immune surveillance and preserves connective tissue organization. All of this constitutes a tumor microenvironment that tumor cells manipulate for tumorogenesis and tumor progression such as fibroblast activation, neovascularization, or secretion of growth factors and chemokines, which mediate numerous physiological and pathological processes related primarily to cell homing and migration, together with others [2, 4].

Chemokines are a family of cytokines with the ability to induce gradient-dependent directional chemotaxis and are secreted by a wide variety of epithelial cells and stromal cells [5–7]. These carry out their biological function through interaction with their transmembrane receptors on their target cells. The C-X-C motif chemokine receptor 4 (CXCR4) is expressed in numerous tissues and is overexpressed in more than 23 different types of human cancers including kidney, lung, brain, prostate, breast, pancreas, ovarian, and melanomas [7–16]. It has been described that its inhibition may alter tumor-stroma interaction and drug sensitization, and also affect tumor growth and metastasis [7]. In addition to contributing to tumor-stroma interaction, CXCR4 is also expressed in a key cell type in the tumor microenvironment, cancer stem cells (CSCs), and in particular it has been proposed as a marker for pancreatic CSCs (PaCSCs), along with CD133 and epithelial cell adhesion molecule (EpCAM), among others [17]. The CXCR4 expression in PaCSCs is associated with increased invasiveness, self-renewal, and survival rates [7, 18, 19].

The CXCR4 receptor is binded by C-X-C motif chemokine 12 (CXCL12), a lymphocyte chemoattractant and regulator of hematopoiesis. The CXCR4/CXCL12 axis plays a considerable role in different biological processes by triggering several signaling pathways (mTOR, AKT, NF-KB, or JAK/STAT). For this reason, it is of special interest in cancer research and it is relevant as a targeted approach [20].

Given the impact of the CXCR4 receptor in the interactions between tumor cells and their microenvironment and its involvement in important signaling pathways in cancer, it is proposed as a very promising prognostic biomarker and as an aim for new targeted therapies in cancer. Although anti-CXCR4 therapies are being developed, the value of CXCR4 overexpression in different tumors (as breast cancer, lung cancer, or pancreatic cancer) remains unclear and several studies have provided a non-significant association between CXCR4 expression and clinical outcome [21].

Due to these reasons, given the significant proportion of stroma found in PDAC, in contrast to other types of cancer, it is of special interest to study the expression of this marker by focusing on its manifestation in the stroma. The vast majority of studies performed to date analyze the expression of this receptor in biopsies, without differentiating the expression in the tumor cells or in the cells that make up their microenvironment. Moreover, despite the large number of studies performed, the actual function of the stroma associated with pancreatic cancer remains largely unknown [22]. Therefore, in this study, we propose to focus on the study of the CXCR4 expression in the stroma to contribute to the knowledge of stromal biology and to determine its prognostic implication in PDAC.

## Methods

### Tissue samples

For prospective analysis of the CXCR4 expression, tissue samples were obtained from surgical specimens with R0 resection of 33 patients who were diagnosed with and treated for PDAC by pathological section analysis from January 2017 to September 2022. The non-tumor tissue (*n* = 23) was obtained from patients with PDAC (*n* = 33). This experimental analytic study was approved by the Clinical Research Ethics Committee of the University General Hospital (Ciudad Real, Spain). Institutional board and informed consent were taken from all individual participants for the use of clinical materials for research purposes.

The clinicopathologic variables were obtained from the medical records and the disease stages of the patients were classified according to the 8th Edition of the American Joint Committee (AJCC) Cancer Staging Manual: Pancreas and Hepatobiliary Cancers [23]. The clinical information for all patients was complete.

### Immunohistochemical analysis and interpretation of results

For immunohistochemistry (IHC) staining clinical specimens were formalin-fixed, paraffin-embedded, and cut into sections with a thickness of 4 µm. The sections were deparaffinized at 65 °C for 60 min in a humified oven and after, exposed to a heat-induced pretreatment for antigen retrieval (pTlink, 20 min, 90 °C). After they were blocked with peroxidase (EnVision™ FLEX Peroxidase-Blocking reagent, cat. no SM801, AutostainerLink 48) and submerged in Target Retrieval Solution (EnVision™ FLEX Target Retrieval Solution, Low pH (50x), AutostainerLink 48), the slides were immunostained with primary antibody mouse anti-human CXCR4 (CXCR4 monoclonal antibody (12G5), cat no. 35–8800, dilution 1:150, Invitrogen, USA). The incubation was carried out at 4 °C for 40 min. The reactions were detected using the EnVision™ Flex/HRP Detection Reagent (Cat. no SM802; AutostainerLink 48) standard polymer technique. In addition, the signal intensity was amplified using EnVision™ Flex + Mouse linker (Cat. no. K8021; AutostainerLink 48). Finally, the slides were developed with DAB + CHROMOGEN (EnVision™ FLEX DAB + Substrate Chromogen cat. no SM803, AutostainerLink 48). All the slides were counterstained with Hematoxylin and Eosin (H&E). For controls formalin-fixed and paraffin-embedded tissue samples of the human tonsil were applied. The number of positively stained cells (0–100%) in non-tumor and tumor pancreatic tissues was scored by 1 pathologist blinded to patient outcome and to all data in the clinicopathological findings. In the first study, we compare non-tumor tissue versus tumor pancreatic tissues (*n* = 23). Each sample was subdivided into the underexpression group if the expression of CXCR4 in the stroma was higher in non-tumor tissue than in PDAC tissue; on the contrary, each sample was subdivided into the overexpression group if the expression of CXCR4 in the stroma was higher in PDAC than non-tumor tissue. For the survival and recurrence analysis, in the second study, we focused only on tumor tissue. A staining percentage resulting under the median was classified as low CXCR4 expression; in opposition to this, a staining percentage resulting over the median was classified as high CXCR4 expression.

### Statistical analysis

Quantitative variables were analyzed using the mean percentage and standard error of the mean (SEM). Qualitative variables were expressed as counts and frequencies, *n* (%). The correlation between the marker’s expression and the clinicopathological factors of patients with pancreatic cancer was analyzed with the *χ*^2^ test and Fisher’s exact test. Cumulative survival rates were calculated by the Kaplan–Meier method, and differences in survival rates were analyzed by the log-rank test. Logistic regression and Spearman’s rank test were used for univariate analysis of CXCR4 with clinicopathological data. *P-*values < 0.05 were considered statistically significant. Statistical analysis was conducted using SPSS 29.0 (IBM SPSS, Armonk, NY, USA) and the graphical representations were displayed with GraphPad Prism 9.5.1 (GraphPad Software, Boston, MA, USA).

## Results

Non-tumor tissues and tumor pancreatic tissues from 23 patients (non-tumor tissue was obtained from 23 of the 33 patients) with PDAC were analyzed for this first study. The percentage of stroma and CXCR4 expression in different cell types revealed varying expression amounts between non-tumor tissue and tumor tissue in 23 patients as depicted in Table 1. The percentage of stroma in non-tumor tissue was lower than in tumor pancreatic tissue (32.4 ± 5.2 *vs* 67.4 ± 4.8; *P*-value = 0.001), and the level of CXCR4 protein expression in stromal cells was lower in non-tumor tissues and higher in tumor pancreatic tissues, with rates of positive expression of CXCR4 being 8.7 ± 4.6 and 23.5 ± 6.1, respectively (*P*-value = 0.022). No significant differences were identified in total cells and inflammatory cells between non-tumor tissue and tumor pancreatic tissue.

**Table 1:** Percentage of stroma and CXCR4 expression in total, inflammatory and stromal cells in non-tumor tissues versus tumor pancreatic tissues (*n* = 23)

**Characteristics**	**Non-tumor tissue** (M ± SEM)	**Tumor tissue** (M ± SEM)	***P*-value**
**Percentage of stroma**	32.4 ± 5.2	67.4 ± 4.8	0.001^*^
**Percentage of CXCR4 in total cells**	28.3 ± 5.6	34.6 ± 4.9	0.217
**Percentage of CXCR4 in inflammatory cells**	17.9 ± 6.8	18.5 ± 5.1	0.755
**Percentage of CXCR4 in stromal cells**	8.7 ± 4.6	23.5 ± 6.1	0.022^*^

*M* mean, *SEM* Standard error of mean

^*^P < 0.05, tumor versus non-tumor tissue

**Fig. 1:**
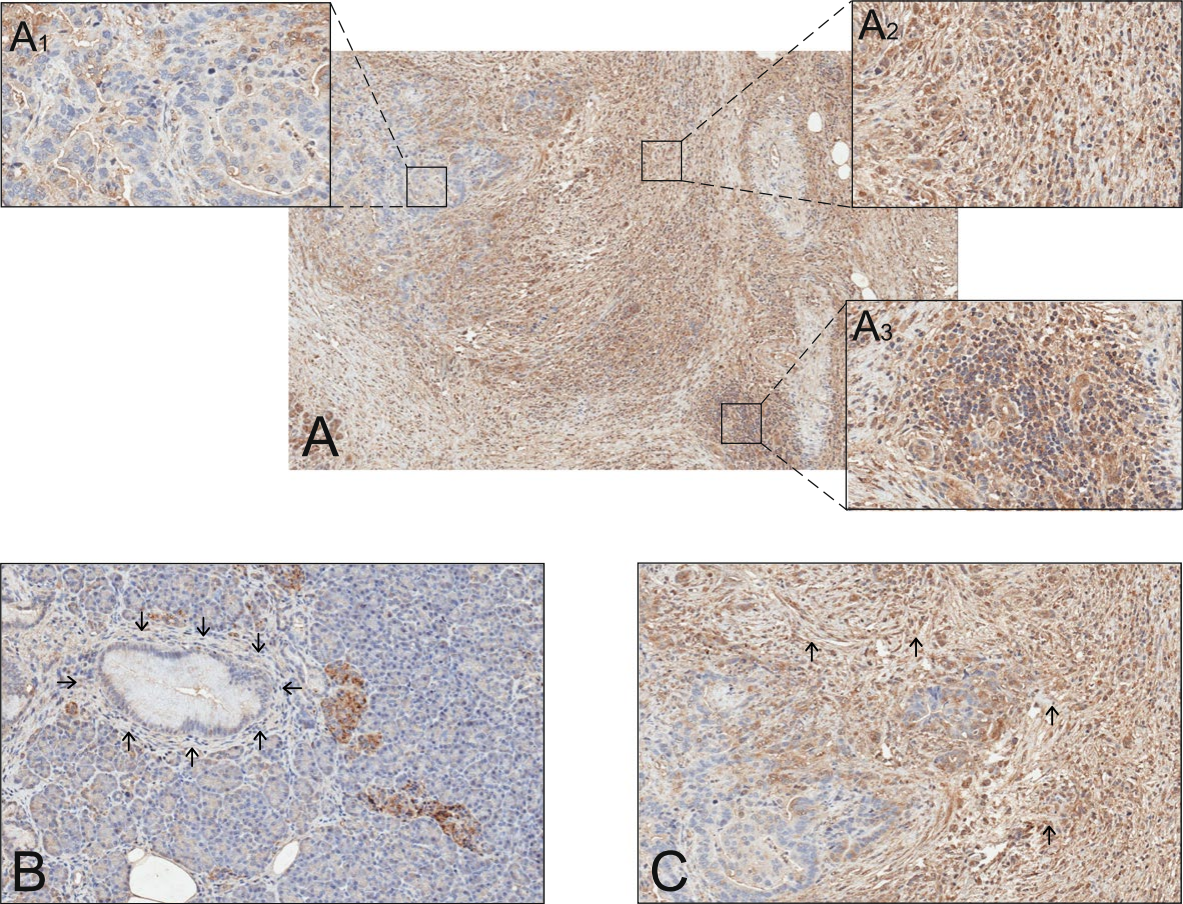
Representative immunohistochemical staining for CXCR4 in non-tumor tissue and tumor pancreatic tissue using hematoxylin and eosin staining. **A** CXCR4 positive cytoplasmic expression in a PDAC tissue; magnification × 8. Inset, the expression of CXCR4 in tumor cells, stromal cells, and inflammatory cells (A1, A2, A3, respectively); magnification × 40; **B** CXCR4 negative cytoplasmic expression in stromal cells in non-tumor tissue; magnification × 17.2; **C** CXCR4 positive cytoplasmic expression in stromal cells in tumor pancreatic tissue; magnification × 17.2. The arrows indicate the stromal cells which show positive or negative staining for CXCR4, respectively

Representative immunohistochemical staining of CXCR4 expression in a human PDAC patient was presented in Fig. 1A; inset, higher magnification demonstrates cytoplasmic CXCR4 being negative in tumor cells, and positive in stromal cells and inflammatory cells (lymphocytes) (Fig. 1A1, 1A2, 1A3, respectively). The staining of non-tumor tissue (Fig. 1B) for CXCR4 revealed a negative cytoplasmic stromal cell expression, with an increase in CXCR4 staining in the cytoplasm of stromal cells in tumor pancreatic tissue (Fig. 1C).

Based on the difference in CXCR4 expression in the stroma in non-tumor tissues compared with tumor pancreatic tissues in these 23 patients, we analyzed the clinicopathological data and the association between underexpression and overexpression profiles of CXCR4 (Table 2). The patients (13 with CXCR4 underexpression and 10 with CXCR4 overexpression) showed no differences in age and gender. The mean follow-up period was 25.0 ± 4.2 months (range, 1–67 months). No association was observed between CXCR4 expression and any of the clinical or pathological data as depicted in Table 2.

**Table 2:** Clinicopathological factors of PDAC patients with non-tumor and tumor pancreatic tissues (*n* = 23) and comparison between CXCR4 underexpression (*n* = 13) versus overexpression (*n* = 10) in stroma

**Characteristics**	**Case** (*n* = 23)	**CXCR4** **underexpression** (*n* = 13)	**CXCR4** **overexpression** (*n* = 10)	***P*-value**
	**M ± SEM**	**M ± SEM**	**M ± SEM**	
**Age (years)**	64.4 ± 2.2	66.8 ± 2.6	61.3 ± 3.7	0.23
**Overall survival (OS, months)**	25.0 ± 4.2	24.5 ± 6.2	25.50 ± 5.6	0.56
**Disease-free survival (DFS, months)**	21.9 ± 4.5	22.1 ± 6.7	21.50 ± 6.1	0.98
**Gender**	***n* (%)**	***n* (%)**	***n* (%)**	
Male	11 (47.8)	6 (46.2)	5 (50.0)	0.99
Female	12 (52.2)	7 (53.8)	5 (50.0)	
**T status**				
T1/T2	20 (87.0)	12 (92.3)	8 (80.0)	0.56
T3/T4	3 (13.0)	1 (7.7)	2 (20.0)	
**N status**				
N0	11 (47.8)	5 (38.5)	6 (60.0)	0.34
N1	10 (43.5)	6 (46.2)	4 (40.0)	
N2	2 (8.7)	2 (15.4)	0 (0.0)	
**M status**				
No	23 (100.0)	13 (100.0)	10 (100.0)	-
**TNM stage**				
I/IIA	10 (43.5)	5 (38.5)	5 (50.0)	0.69
IIB/III	13 (56.5)	8 (61.5)	5 (50.0)	
**Tumor differentiation**				
Well/moderate	18 (78.3)	11 (84.6)	7 (70.0)	0.62
Poor	5 (21.7)	2 (15.4)	3 (30.0)	
**Tumor location**				
Head	16 (69.6)	9 (69.2)	7 (70.0)	0.99
Body/tail	7 (30.4)	4 (30.8)	3 (30.0)	
**Vascular invasion**				
Absent	15 (65.2)	9 (69.2)	6 (60.0)	0.69
Present	8 (34.8)	4 (30.8)	4 (40.0)	
**Lymphatic invasion**				
Absent	14 (60.9)	7 (53.8)	7 (70.0)	0.69
Present	9 (39.1)	6 (46.2)	3 (30.0)	
**Neural invasion**				
Absent	4 (17.4)	3 (23.1)	1 (10.0)	0.60
Present	19 (82.6)	10 (76.9)	9 (90.0)	
**Local recurrence**				
Absent	16 (69.6)	8 (61.5)	8 (80.0)	0.40
Present	7 (30.4)	5 (38.5)	2 (20.0)	
**Distant metastasis**				
Absent	13 (56.5)	7 (53.8)	6 (60.0)	0.99
Present	10 (43.5)	6 (46.2)	4 (40.0)	
**Exitus**				
No	11 (47.8)	6 (46.2)	5 (50.0)	0.99
Yes	12 (52.2)	7 (53.8)	5 (50.0)	

*CXCR4* C-X-C motif chemokine receptor 4, *TNM* Tumor node metastasis, *M* mean, *SEM* Standard error of mean

**Fig. 2:**
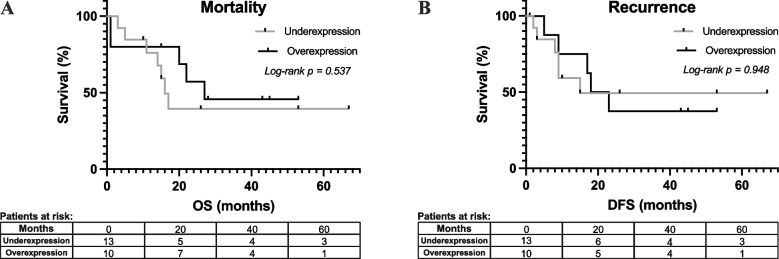
Mortality and recurrence Kaplan–Meier analysis of CXCR4 underexpression (*n* = 13) versus overexpression (*n* = 10) in stroma. **A** Overall survival (OS). **B** Disease-free survival (DFS)

Mortality and recurrence rates among underexpressed and overexpressed CXCR4 patients were studied using a Kaplan–Meier model (Fig. 2). Overall survival (OS) analysis showed a median of 16.0 months (range, 3–67 months) for CXCR4 underexpression patients versus 27.0 months (range, 1–53 months) for CXCR4 overexpression patients. No significant differences were found (log-rank* p* = 0.537; Fig. 2A). In the disease-free survival (DFS) analysis, a median of 15.0 months (range, 2–67 months) for CXCR4 underexpression patients versus 20.5 months (range, 1–53 months) for CXCR4 overexpression patients was observed. No significant differences were found (log-rank* p* = 0.948; Fig. 2B).

In addition, in order to investigate the role of CXCR4 in the progression of pancreatic cancer, the present study also analyzed the association between the expression profiles of CXCR4 and clinicopathological factors only in tumor tissues from 33 patients with PDAC (Table 3). The patients consisted of 16 males and 17 females, with a mean age of 64.8 ± 1.7 years (range, 42–78 years). The mean follow-up period was 21.0 ± 3.2 months (range, 1–67 months). Of the 33 patients, 25 (75.8%) received adjuvant treatment. Adjuvant therapy had no impact on survival. The CXCR4 expression was associated with tumor differentiation (*P*-value = 0.05). However, no correlation was observed between the CXCR4 expression and the rest of the clinicopathological factors as illustrated in Table 3. Furthermore, logistic regression analysis revealed that for each increasing unit in the percentage of CXCR4 expression in the stromal cells, the probability of poor differentiation increases by 2.7%, OR = 1.027 (1.000–1.054); *P*-value = 0.05. However, the rest of the clinicopathological factors failed to reach significance.

**Table 3:** Clinicopathological factors of PDAC patients with tumor tissues (*n* = 33) and the association with CXCR4 expression in stroma (*n* = 33)

**Characteristics**	**Case** (*n* = 33)	**CXCR4** **expression**	***P*-value**
	**M ± SEM**	***ρ* Spearman**	
**Age (years)**	64.8 ± 1.7	− 0.353^a^	0.08
**Overall survival (OS, months)**	21.0 ± 3.2	− 0.055^a^	0.76
**Disease-free survival (DFS, months)**	18.2 ± 3.3	− 0.133^a^	0.46
**Gender**	***n* (%)**	**M ± SEM**	
Male	16 (48.5)	20.0 ± 4.7	0.84
Female	17 (51.5)	31.2 ± 9.2	
**T status**			
T1/T2	29 (87.9)	25.9 ± 5.9	0.73
T3/T4	4 (12.1)	25.0 ± 10.4	
**N status**			
N0	13 (39.4)	35.4 ± 9.8	0.09
N1	14 (42.4)	25.7 ± 7.2	
N2	6 (18.2)	5.0 ± 5.0	
**M status**			
No	33 (100.0)	25.8 ± 5.3	-
**TNM** **stage**			
I/IIA	12 (36.4)	34.2 ± 10.3	0.23
IIB/III	21 (63.6)	21.0 ± 5.8	
**Tumor** **differentiation**			
Well/moderate	24 (72.7)	19.2 ± 5.3	0.05^*^
Poor	9 (27.3)	43.3 ± 11.5	
**Tumor** **location**			
Head	23 (69.7)	30.0 ± 6.7	0.18
Body or tail	10 (30.3)	16.0 ± 7.3	
**Vascular** **Invasion**			
Absent	21 (63.6)	24.8 ± 6.8	0.77
Present	12 (36.4)	27.5 ± 8.5	
**Lymphatic** **invasion**			
Absent	20 (60.6)	24.0 ± 6.7	0.68
Present	13 (39.4)	28.5 ± 8.8	
**Neural invasion**			
Absent	6 (18.2)	36.7 ± 15.8	0.48
Present	27 (81.8)	23.3 ± 5.4	
**Local recurrence**			
Absent	26 (78.8)	30.0 ± 6.3	0.22
Present	7 (21.2)	10.0 ± 4.4	
**Distant metastasis**			
Absent	18 (54.5)	22.2 ± 6.4	0.44
Present	15 (45.5)	30.0 ± 8.8	
**Exitus**			
No	15 (45.5)	20.0 ± 7.4	0.15
Yes	18 (54.5)	30.6 ± 7.4	

*CXCR4* C–X–C motif chemokine receptor 4, *TNM* Tumor node metastasis, *M* Mean, *SEM* Standard error of mean

^a^Spearman’s correlation coefficient. All other comparisons in the table are Mann–Whitney *U* or Kruskal Wallis tests. *P < 0.05

**Fig. 3:**
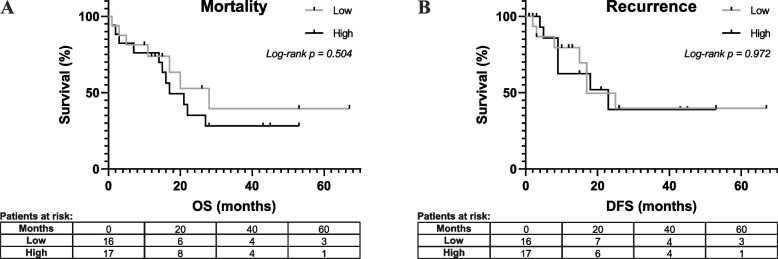
Mortality and recurrence Kaplan–Meier analysis of low CXCR4 expression (*n* = 16) versus high CXCR4 expression (*n* = 17) in stroma in PDAC patients. **A** Overall survival (OS). **B** Disease-free survival (DFS)

Mortality and recurrence rates among low and high CXCR4 expression were studied using a Kaplan–Meier model in PDAC patients (Fig. 3). Overall survival (OS) analysis showed a median of 28.0 months (range, 1–67 months) for low CXCR4 expression versus 17.0 months (range, 1–53 months) for high CXCR4 expression in PDAC patients. No significant differences were found (log-rank* p* = 0.504; Fig. 3A). In the disease-free survival (DFS) analysis, a median of 17.0 months (range, 1–67 months) for low CXCR4 expression patients versus 23 months (range, 1–53 months) for high CXCR4 expression in PDAC patients was observed. No significant differences were found (log-rank* p* = 0.972; Fig. 3B).

## Discussion

Despite improvements in diagnostic techniques, surgical procedures, and cancer therapies, the prognosis of patients with PDAC remains extremely poor, and further studies are needed to higher knowledge of tumor pathogenesis and to lead to new preventive and therapeutic options. In recent years, special attention is being paid to the role of tumor-associated stroma as it plays an important part in tumor development and progression, as well as, resistance to therapies [22]. The role of the CXCR4 receptor is key to the interaction of cancer cells and their microenvironment and it is considered a promising prognostic biomarker for cancer. It is expressed in numerous cell types, but little is known about the relationship between its expression in the stroma and its prognostic implication. Its value in pancreatic cancer is yet to be determined, and it is known that PDAC contains a very important tumor-bound stromal component, so elucidating the relationship of the CXCR4 expression in the stroma and its involvement in PDAC is of particular interest.

### Importance of stroma in PDAC

In the present study, we first compared the percentage of tumor-associated stroma with non-tumor stroma tissue in 23 patients diagnosed with PDAC. It was observed that the percentage of stroma in the total volume was significantly higher (*P*-value = 0.001) in tumor tissue than in non-tumor tissue samples (67.4 ± 4.8 and 32.4 ± 5.2, respectively), which may indicate that the stroma might be involved in the development of pancreatic cancer (Table 1). It has been proposed that neoplastic cells and extracellular matrix interactions stimulate a large desmoplastic reaction and that the stroma production is promoted by the activation of multiple neoplastic cell-derived signaling pathways. Furthermore, numerous studies have provided evidence that the microenvironment co-evolves with transformed epithelial cells in different carcinomas [24]. This is consistent with our findings, in which the percentage of stroma in the tumor samples is quite high as has been found in other research projects [2, 22, 24, 25].

### Expression of CXCR4 as a biomarker of PDAC

The chemokine CXCR4 receptor has been related with tumor-stroma interactions, and its expression has been detected in different microenvironment cells [3], in CSCs [17, 26], tumor cells [27], or in cells of the immune system such as naive T cells, some memory T cells, B cells, and mature dendritic cells. These play a central role in lymphocyte trafficking and homing to lymph nodes [28]. Once we proved the fundamental role of the stroma in tumor samples, we decided to analyze the CXCR4 expression in different cell components of our samples (Table 1).

All samples had cytoplasmic expression of CXCR4. This is consistent with the results of Wehler et al., in which CXCR4 staining occurred predominantly in the cell cytoplasm, being scarcely found in the membrane in some of those cases, and in alignment with the description of an inducible translocation of CXCR4 from the membrane to the cytoplasm [27, 29].

As depicted in Table 1, on the one hand, as in the data series, the overall expression of CXCR4 was studied; on the other hand, the percentage of CXCR4 expression was only analyzed in the stroma of the samples, and the CXCR4 expression found in the cells of the immune system was also analyzed. The CXCR4 expression was significantly higher (*P*-value = 0.022) in the stroma of tumor tissues than in the stroma of non-tumor tissues (23.5 ± 6.1 and 8.7 ± 4.6, respectively), whereas no statistical association was possible in the evaluation of expression in tissues globally or in immune cells. The data set described here constitutes a relatively small set of samples to be analyzed compared to other larger data sets of CXCR4 expression studies in PDAC. These studies analyze the whole tissue [28], so we propose to focus the analysis on the CXCR4 expression in the stroma and eliminate possible artifacts that may be generating CXCR4 expression in other cellular subtypes.

### Association of the CXCR4 expression in the stroma with patient characteristics

In order to determine the role of the CXCR4 stroma expression in the pathology of PDAC, the present study also analyzed the association between overexpression or underexpression of CXCR4 in tumor tissues versus their matched non-tumor tissue samples with clinicopathological factors, but we found no statistical significance (Table 2). This happened in contrast to other datasets such as the studies by Darash-Yahana et al., which positively correlate the CXCR4 expression with tumor growth, vascularization, and metastasis in other cancer types such as prostate cancer [4]. We also did not find an association between mortality or recurrence like Kure et al., probably due to the small sample size (Fig. 2) [30].

### Relevance of the CXCR4 expression in tumor stroma in PDAC differentiation

Focusing exclusively on tumor tissues of PDAC patients, we evaluated the role of the CXCR4 expression in tumor stroma with respect to prognostic factors, finding a relationship between poor differentiation and high CXCR4 expression (Table 3). Studies in glioblastoma (GBM) suggest an important role of CXCR4 in cell proliferation and in maintaining the neoplastic phenotype of GBM cells [12]. Therefore, proliferative activity and loss of original conformation, mediated by CXCR4, could promote poor tumor differentiation. In addition, abnormalities affecting signal transduction pathways involved in the control of cell growth and other malignant properties may be affected by CXCR4. These abnormalities might compromise tumor staging and the degree of differentiation [28]. Indeed, Heinrich et al. observe increased CXCR4-mediated proliferation in pancreatic cell lines [31].

There is controversy about the prognostic value of CXCR4 overexpression in different tumors, and although there are numerous related studies, Zhao et al. provided a negligible association between CXCR4 and clinical outcome in some tumor types such as breast cancer, lung cancer, or pancreatic cancer [21]. There are numerous series of data correlating CXCR4 overexpression with poor prognosis in pancreatic cancer. Zhan et al. found no association between poor differentiation and CXCR4 overexpression, but did find an association with pathological type, lymph node stage, and TNM stage [32]. In experiments carried out in mice, Malik et al. linked the CXCR4/CXCL12 axis to PDAC growth, spread, chemoresistance, and immune evasion [2]. On the other hand, Kure et al. found a high level of correlation between the CXCR4 expression with well-differentiated PDAC [30]. As Gebauer et al., no association with mortality or recurrence was found [33]. All this previous research has analyzed the sample globally, so it would be interesting to focus the study on the stroma, which could reduce the heterogeneity of the results.

## Limitations

As this is a low-incidence pathology and the study was carried out in a relatively small hospital, the number of samples is less than desired. Studies with larger samples would be necessary to validate the results. Significance has been found in the expression of CXCR4 in the stroma. Increasing the sample size would help to define CXCR4 as a potential biomarker, and locate the specific area of analysis. This could eliminate possible artifacts that are responsible for the variability of the results obtained in the studies already published.

For this study, only the immunohistochemistry technique has been developed. It would be interesting to have accompanied it with another method to validate the results obtained, but the difficulty of being able to separate stromal cells from other cell subtypes in the tissues analyzed prevents the use of other techniques such as Real-Time PCR or Western blot. These techniques would not allow to explore the expression levels of CXCR4 in the stroma without the sample being distorted by the expression of the protein in other cell subtypes, such as cells of the immune system or tumor cells.

## Conclusions

Numerous studies have addressed the analysis of CXCR4 receptor expression in various types of cancer, and many relate it to poor prognosis, although there is no clear consensus on its effect on the clinical outcome of PDAC patients. It is well known that the stroma is not simply a structural component but constitutes a fundamental microenvironment for tumor development and progression. In this study, we reflect on the importance of focusing on the CXCR4 expression analysis in the stroma of PDAC-diagnosed patients, due to the high expression of CXCR4 in the tumor stroma. Although we observed an association between CXCR4 and poor tumor differentiation, no association was found between CXCR4 expression and overall and disease-free survival. Neither did we find an association with the rest of the clinicopathological variables. Taking into account the previous considerations, more research will be needed to help us understand the role of this receptor in the tumor stroma and its possible implication in the prognosis of this deadly cancer.

## Data Availability

The data relating to the study were obtained from the Department of Surgery of the University General Hospital of Ciudad Real and were provided by the Biobank of the University General Hospital of Ciudad Real. Data supporting the conclusions of this study are available from the corresponding author on reasonable request.

## Abbreviations

AJCC: American Joint Committee
CSCs: Cancer stem cells
CXCR4: C–X–C motif chemokine receptor 4
CXCL12: C–X–C motif chemokine 12
EpCAM: Epithelial cell adhesion molecule
GBM: Glioblastoma
H&E: Hematoxylin and eosin
IHC: Immunohistochemistry
PaCSCs: Pancreatic cancer stem cells
PDAC: Pancreatic ductal adenocarcinoma
